# The Medical Record Visiting List or Physician’s Diary for 1884

**Published:** 1884-01

**Authors:** 


**The Medical Record Visiting List or Physician's Diary for 1884.** New York: Wm. Wood & Co.

This is certainly the handsomest of the many diaries offered to the profession, and not a few think it is also the most convenient in its arrangement. The new book, for 1884, is out, and we would advise our friends to look at it before buying any other.

